# Conserved molecular structure of the centromeric histone CENH3 in *Secale* and its phylogenetic relationships

**DOI:** 10.1038/s41598-017-17932-8

**Published:** 2017-12-15

**Authors:** E. V. Evtushenko, E. A. Elisafenko, S. S. Gatzkaya, Y. A. Lipikhina, A. Houben, A. V. Vershinin

**Affiliations:** 1 Institute of Molecular and Cellular Biology SB RAS, Novosibirsk, 630090 Russia; 2 Institute of Cytology and Genetics SB RAS, Novosibirsk, 630090 Russia; 3 Leibniz Institute of Plant Genetics and Crop Plant Research (IPK), Gatersleben, 06466 Stadt Seeland Germany

## Abstract

It has been repeatedly demonstrated that the centromere-specific histone H3 (CENH3), a key component of the centromere, shows considerable variability between species within taxa. We determined the molecular structure and phylogenetic relationships of CENH3 in 11 *Secale* species and subspecies that possess distinct pollination systems and are adapted to a wide range of abiotic and biotic stresses. The rye (*Secale cereale*) genome encodes two paralogous *CENH3* genes, which differ in intron-exon structure and are transcribed into two main forms of the protein, αCENH3 and βCENH3. These two forms differ in size and amino acid substitutions. In contrast to the reported differences in CENH3 structure between species within other taxa, the main forms of this protein in *Secale* species and subspecies have a nearly identical structure except some nonsynonymous substitutions. The CENH3 proteins are strictly controlled by genetic factors responsible for purifying selection. A comparison between *Hordeum*, *Secale* and *Triticum* species demonstrates that the structure of CENH3 in the subtribes Hordeinae and Triticinae evolved at different rates. The assumption that reticulate evolution served as a factor stabilizing the structure and evolutionary rate of CENH3 and that this factor was more powerful within *Secale* and *Triticum* than in *Hordeum*, is discussed.

## Introduction

The pivotal role in the proper chromosome segregation during meiosis and mitosis lies with centromeres. In most species the centromere identity is defined by the presence of the centromere-specific variant of histone H3 known in plants as centromere-specific histone H3 variant CENH3 (for review, see^1,2^). Any error in transcription, translation, modification or import can affect the assembly of intact CENH3 chromatin, which would result in the loss of CENH3 from the centromeres and hence in the centromere identity (reviewed in^3^). In contrast to the conserved structure of canonical histone Н3, CENH3 shows considerable variability across species^4,5^. Different domains of this molecule evolved differently. An extended N-terminal tail (NTT) and loop 1 of the histone fold domain (HFD) putatively interact with centromeric DNA^6^ and show signatures of positive selection in some animal and plant species^7,8^, while the part of the HFD domain outside loop 1 is generally conserved^8–10^.

Most of the diploid plant species (*Arabidopsis thaliana*, maize and rice), in which the structure and copy number of CENH3 have been determined, have this gene as a single copy^8,11,12^. However, some species in the Triticeae tribe have CENH3 in two variants. They are tetraploid and diploid wheat (*Triticum*) species^13^, diploid barley (*Hordeum*) species^14^ and *Aegilops* species^13^. The levels of expression of these two CENH3 variants and the efficiency of their incorporation at centromeres vary across different tissues as demonstrated for barley^15^ and between wild and cultivated tetraploid wheats, which is considered as a signature of adaptive evolution^13^.

Rye (*Secale*) is a small but important genus of the Triticeae tribe adapted to a wider range of environmental and climatic conditions than wheat or barley^16^. Cultivated, weedy and wild species in *Secale* have different pollination systems (self-incompatible, allogamous vs self-compatible, autogamous) and life-cycle durations (perennials vs annuals). Sencer & Hawkes^17^ classified this genus as consisting of three biological species: the outcrossing perennial *S*. *strictum* Presl., the outcrossing annual *S*. *cereale* L., and the autogamous annual *S*. *sylvestre* Host. This classification received further support from morphometrical data^18^ and molecular analysis^19^. Traditional rye varieties are panmictic populations displaying high levels of heterozygosity and heterogeneity^20^, which might have resulted from outcrossing pollination and facilitated interspecies hybridization. Because the CENH3 proteins and genes encoding them in *Secale* species have yet to be known, it is intriguing to explore the molecular structure and the evolutionary dynamics of this central component of centromere specification and function.

We have identified and characterized CENH3 variants in *Secale* species and subspecies, the intron-exon structure of the *CENH3* genes and their phylogenetic relationships in *Secale* and closely related genera, *Triticum* and *Hordeum*, in Triticeae. We found that CENH3 sequences in *Secale* species and subspecies have a nearly identical structure except some nonsynonymous substitutions. This implies that the general view about rapidly evolving CENH3s is not universal – at least, it does not apply to the genus *Secale*. A comparison of *Hordeum*, *Secale* and *Triticum* species demonstrated that the CENH3 structure in the subtribes Hordeinae and Triticinae (the latter including *Triticum* and *Secale* species^21^) evolved at different rates. We hypothesize that past remote hybridization events (reticulate evolution) served as a factor stabilizing the structure of the CENH3 genes and proteins and that this factor was more powerful within *Secale* and *Triticum* than it was in the other cereals taxa, including *Hordeum*.

## Results

### Identification and characterization of the CENH3 forms in Secale

**Figure 1:**
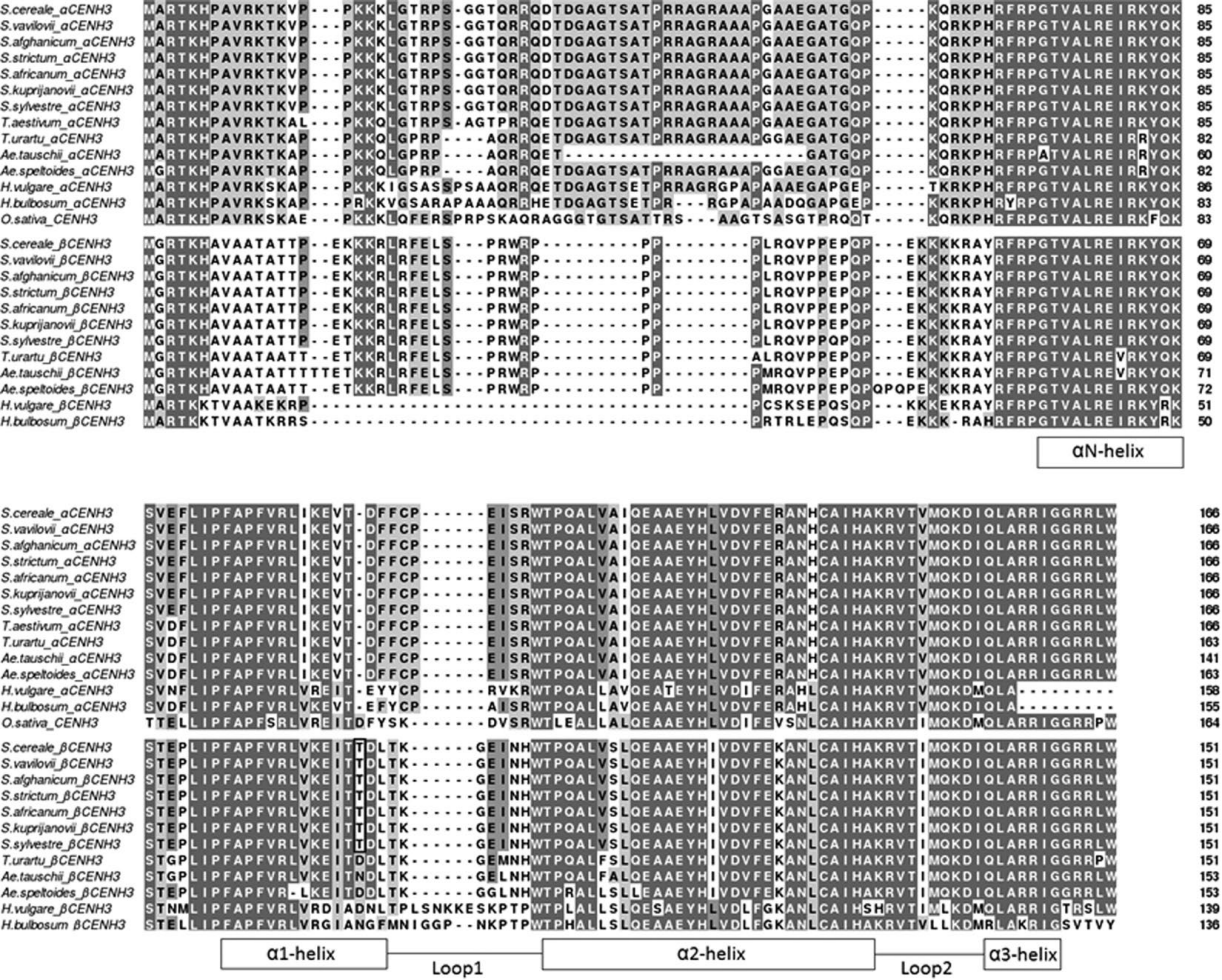
Multiple alignment of the amino acid sequences of CENH3 proteins. αCENH3-v1 and βCENH3-v1 from rye accessions are aligned with CENH3 proteins in *Triticum*, *Aegilops*, *Hordeum*, and *Oryza sativa* accessions: αCENH3 of *T*. *aestivum* (JF969285.1), *T*. *urartu* (KM507181.1), *A*. *tauschii* (KM507183.1), *A*. *speltoides* (KM507182.1), *H*. *vulgare* (JF419328.1), *H*. *bulbosum* (GU245882.1), *O*. *sativa* (AY438639.1) and βCENH3 of *T*. *urartu* (KM507184.1), *A*. *tauschii* (KM507186.1), *A*. *speltoides* (KM507185.1), *H*. *vulgare* (JF419329.1), *H*. *bulbosum* (JF419330.1). For convenience, alpha and beta forms are grouped into two separate blocks. Separate HFD regions are singled out according to^27^. The amino acid threonine that occurs in the βCENH3 HFD, but not in the αCENH3 HFD, is framed. Amino acid residues identical in all species are shaded in dark gray.

We searched the NCBI SRA database for *CENH3* of *S*. *cereale* and found partial sequences with homology to *αCENH3* of *H*. *vulgare* and *βCENH3* of *T*. *urartu*. Based on these, PCR primers were designed and used for amplifying complete CENH3 transcripts of rye. After cloning of PCR products and sequencing of randomly selected clones the presence of two main forms of CENH3 (called αScCENH3 and βScCENH3) were revealed in rye. The α*ScCENH3* sequence is 501 bp in length and the deduced protein is made up of 166 amino acids. In *S*. *cereale*, β*CENH3* is distinct from α*CENH3* in that the former has several deletions in the N-terminal tail (NTT) and the insertion of three nucleotides, АСС, which encode the amino acid threonine (framed in Fig. 1), in the histone fold domain (HFD). Thus, βSc*CENH3* has an overall length of 456 bp and encodes a protein made up by 151 amino acids. Most of the amino acid sequences of the NTT in α*CENH3* and β*CENH3* do not align well with each other (Fig. 1). The average nucleotide identity between α*CENH3* and β*CENH3* is 81–83%. In the HFD the main differences are concentrated in the α1-helix and loop 1, that is, in the centromere-targeting domain (CATD).

**Figure 2:**
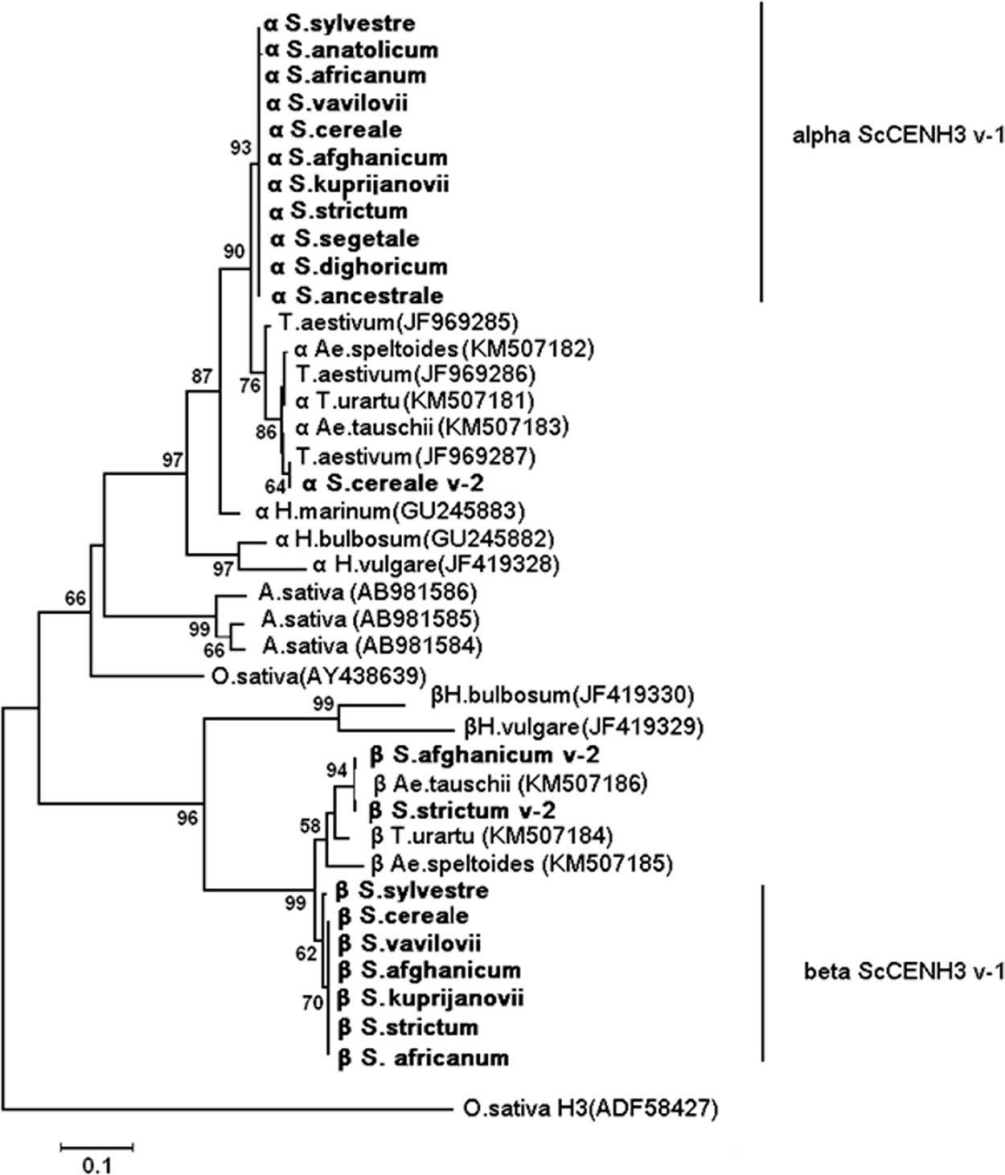
Phylogenetic tree of the deduced CENH3 proteins. Phylogenetic tree inferred using the JTT + G models (measures distances) and bootstrapping (1000 replicates). Bootstrap values are indicated on the branches. NCBI accession numbers are given in parentheses.

**Figure 3:**
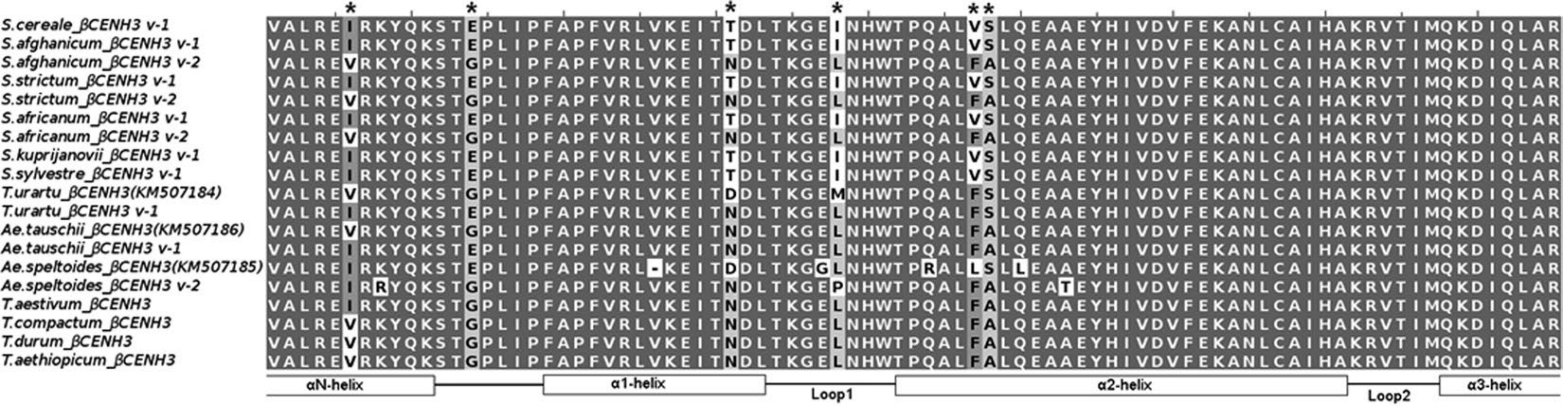
Multiple alignment of the amino acid sequences of βHFDs in Triticeae species. Asterisks indicate polymorphic sites.

In addition to the clones with 501-bp long sequences (αSc*CENH3*-v1), which were the most frequently occurring in the pool of the α*CENH3* clones randomly selected for sequencing, we found clones with shorter inserts, 492 bp in length (Supplementary Fig. S1), with 94% nucleotide identity to αSc*CENH3*-v1 and also with 99% nucleotide identity to one of the *CENH3* sequences identified previously in the genome of *T*. *aestivum* (JF969287.1) and to α*CENH3* in the genome of *T*. *urartu* (KM507181.1). Beside different lengths, they also contain different amino acids at the same positions in different αScCENH3 clones and thus probably reflect individual sequence differences. The shorter variant should be designated as minor, *αScCENH3-*v2, because the percentage of these clones in the pool is low. The highest frequency of αSc*CENH3*-v2 is 18%, which is in the annual *S*. *cereale* ssp. *cereale* (*S*. *cereale* throughout) cv. Otello.

βScCENH3, too, occurs in two variants. The two βScCENH3 variants differ by 14 amino acid substitutions, of which nine are nonsynonymous and three of these are found in loop 1 and α2-helix (Supplementary Fig. S1). β*CENH3-v1* is 456 bp in length and has 95% nucleotide identity to *T*. *urartu* (KM507184.1). β*CENH3-v2* has 95% nucleotide identity to β*CENH3-v1*, 99% nucleotide identity with the *βCENH3* of *A*. *tauschii* (KM507186.1) and is 6 bp longer than β*CENH3-v1*.

Ten additional *Secale* species and subspecies that possess distinct pollination systems and are adapted to a wide range of abiotic and biotic stresses were included in this study. *αCENH3-*v2 was only in five *Secale* accessions and β*CENH3-v2* only in four (Table 1). The frequency of *αCENH3-*v2 is 8–10% in the perennial self-pollinated *S*. *strictum* ssp. *africanum* (*S*. *africanum* throughout) and the annual cross-pollinated *S*. *cereale* ssp. *dighoricum* (*S*. *dighoricum* throughout) (Table 1). β*CENH3-v2* is present in cross-pollinated subspecies: the annual *S*. *cereale ssp*. *afghanicum* (*S*. *afghanicum* throughout), in which it makes up 60% of the clones sequenced, and the perennial *S*. *strictum ssp*. *strictum* (*S*. *strictum* throughout) with 45%. However, it is important to take into account the fact that the non-observation of any rare variant in PCR products for cDNA does not necessarily mean that this variant is not present in the genome. Thus, rye species and subspecies possess their own genus-specific alpha and beta forms of CENH3, as well as variants of these forms, which are also present in *Triticum* and *Aegilops* species (Table 1).

**Тable 1:** List and description of species used.

№	Species	Accession no.	Ploidy/genomic composition	Growth habit	CENH3 variants
1	*S*. *cereale* subsp. *cereale* ‘Otello’	R1264	2x/RR	A,O	α-v1, α-v2, β-v1
2	*S*. *cereale* subsp. *cereale* ‘Black Winter’	9395	2x/RR	A,O	α-v1, β-v1*
3	*S*. *cereale* subsp. *cereale* ‘Imperial’	9368	2X/RR	A,O	α-v1, α-v2, β-v1, β-v2
4	*S*. *cereale* subsp. *vavilovii*	R1027	2x/RR	A,S	α-v1, β-v1
5	*S*. *cereale* subsp. *dighoricum*	R803	2x/RR	A,O	α-v1, α-v2, β-v1*
6	*S*. *cereale* subsp. *ancestrale*	R62	2x/RR	A,O	α-v1, β-v1*
7	*S*. *cereale* subsp. *segetale*	PI 102	2x/RR	A, O	α-v1, β-v1*
8	*S*. *cereale* subsp. *afghanicum*	HR566/86	2x/RR	A,O	α-v1, β-v1, β-v2
9	*S*. *strictum* subsp. *kuprijanovii*	R549	2x/RR	P,O	α-v1, β-v1
10	*S*. *strictum* subsp. *anatolicum*	PI 206992	2x/RR	P,O	α-v1, α-v2, β-v1*
11	*S*. *strictum* subsp. *africanum*	10289	2x/RR	P,S	α-v1, α-v2, β-v1, β-v2
12	*S*. *strictum* subsp. *strictum*	10736	2x/RR	P,O	α-v1, β-v1, β-v2
13	*Secale sylvestre*	R1116	2x/RR	A,S	α-v1, β-v1
14	*Aegilops speltoides*	IG 48993	2x/SS≈BB	A,O	α, β-v1, β-v2
15	*Aegilops tauschii*	IG 46798	2x/DD	A,S	α, β-v1, β-v2
16	*Triticum urartu* Tumanian ex Gandilyan	PI 428183	2x/AA	A,S	α, β-v1, β-v2
17	*T*. *turgidum* L. subsp. dicoccum**	PI 273979	4x/AABB	A,S	α, β
19	*T*. aethiopicum**	TRI14805	4x/AABB	A,S	α, β
19	*T*. *aestivum* L. subsp. aestivum**	TR201	6x/AABBDD	A,S	α, β
20	*T*. *aestivum* L. susp. compactum**	TR189	6x/AABBDD	A,S	α, β

Note: A – annual, P – perennial, O – open-pollinated, S – self-pollinated; Cv – cultivar. β* - subspecies not examined for βCENH3-v2. ** - accessions not examined for CENH3 variants.

Transcripts with the characteristics of the main forms of *CENH3* were found in all the 11 rye species and subspecies analyzed (the rye CENH3 forms given in Fig. 1 are actually αCENH3-v1 and βCENH3-v1). The nucleotide identity of α*CENH3* and β*CENH3* sequences between *Secale* species and subspecies is 98–100%. Deletions in the NTT of β*CENH3* in the rye species and subspecies are noted for having fixed lengths, these lengths being exactly the same as those of deletions in *T*. *urartu* and other donors of the hexaploid wheat genome, *A*. *tauschii* and *A*. *speltoides*. Surprisingly, the structure of this region in the rye species is closer to that in *T*. *aestivum* than to that of αCENH3 in *T*. *aestivum* progenitors. A high level of similarity in the nucleotide sequences of the *CENH3* genes between allopolyploid wheats and various rye species and subspecies, which is 96–97% for *αCENH3*, is reflected by a high level of similarity in their amino acid sequences. A comparison of the αCENH3 sequences in four *S*. *cereale* cultivars (Otello, Black Winter, Imperial and Korotkostebelny 69) with their counterpart in *T*. *aestivum* cv. Chinese Spring (JF969285.1) revealed as few as six amino acid substitutions in the NTT and one in the HFD. In contrast to the close similarities in CENH3 sequences between the rye and wheat species, the structures of CENH3 have considerable differences between the barley species *H*. *vulgare* and *H*. *bulbosum* (JF419329.1 and JF419330.1). Compared to *H*. *bulbosum* αHbCENH3, *H*. *vulgare* αHvCENH3 contains 10 amino acid substitutions in the HFD, largely in loop 1, and three additional amino acids in the NTT. The differences are especially high between the beta forms of CENH3. βHvCENH3 is distinct from βHbCENH3 in that it has 30 nonsynonymous amino acid substitutions throughout the molecule and four additional amino acids. Compared to the beta forms of rye and wheat CENH3, those of barley have longer deletions in the NTT, and this accounts for the differences in the size of this domain: 108 bp in *H*. *bulbosum*, 111 bp in *H*. *vulgare*, and 165 bp in *S*. *cereale*. The mean pairwise distance between the αCENH3 paralogs of *S*. *cereale* and *H*. *vulgare* is 0.122 at nucleotide level and 0.269 at amino acid level; that between *S*. *cereale* and *H*. *bulbosum*, 0.097 and 0.221, correspondingly; and that between *S*. *cereale* and *T*. *aestivum*, 0.033 and 0.043, correspondingly. This comparison shows that both main forms of CENH3 have a surprisingly high structural similarity between *Secale*, *Triticum* and *Aegilops* species, but it is different in barley.

### Phylogeny of rye CENH3

With the Neighbor Joining (NJ) algorithm, a phylogenetic tree was constructed for the amino acid sequences of CENH3 in 11 accessions of rye, the closest rye relatives within Triticeae (wheat, barley and *Aegilops* species), and some monocotyledonous species as well as for the sequence of canonical histone H3 of *O*. *sativa* as an outgroup (Fig. 2). The first node is where two major clusters arise from, one with alpha forms of CENH3 and another with beta forms. In all *Secale* accessions analyzed, αCENH3-v1s fall in the same domain within the cluster. However, the second variant of rye αCENH3, αCENH3-v2, is in another domain, together with the wheat and *Aegilops* accessions. Of the other Triticeae species, the closest to rye αCENH3 was *T*. *aestivum* CENH3 (93–97% nucleotide identity, p (pair-wise distance between orthologs) = 0.040). For other cereal species, *A*. *sativa* and *O*. *sativa*, the corresponding values varied from 78% to 73% and from 0.365 to 0.469.

Both βCENH3 variants form the second major cluster, together with beta forms in the *Aegilops* species and *T*. *urartu*. The alpha and beta forms of the barley species are in the major tree clusters together with their rye, wheat and *Aegilops* counterparts. The CENH3 sequence of *O*. *sativa* forms a separate branch and is in the same major cluster as the alpha forms of Triticeae species.

### Divergence of rye CENH3s

A comparison of NTT and HFD sequences done using the McDonald—Kreitman test^22^ in the subspecies within *S*. *cereale* and *S*. *strictum* revealed a lack of “fixed divergence”, as McDonald and Kreitman put it. We aligned the sequences of all subspecies of each of the given species in one data set and estimated K_a_/K_s_ ratios (also denoted as ω). For both domains, the ratio of nonsynonymous (K_a_) to synonymous substitutions (K_s_) in the alpha and beta forms between species is significantly less than 1 (Table 2), which appears to be a signature of stabilizing selections. To identify potential sites under positive selection, we estimated ω between subspecies. The results of the pair-wise comparisons of subspecies include a few ω >1 instances (one such comparison is given in Supplementary Table S1). Although the difference between the ω value and 1 was not statistically significant in any of these instances, it suggests that within species divergence has signatures of positive selection. Noteworthy, the ω value was higher in the NTT than in the HFD (Table 2), suggesting that the NTT has evolved faster than the HFD. Similarly, the ω values for the beta forms of CENH3 are in all cases higher than those for the alpha forms.

**Тable 2:** Nonsynonymous (Ka) to synonymous (K_s_) nucleotide substitutions in the CENH3s of *Secale* species.

(A)				
	αCENH3 *S*. *strictum*		αCENH3 *S*. *sylvestre*	
	NTT	HFD	NTT	HFD
αCENH3 *S*.*cereale*	0.0081/0.0265 **0.309**	0.0051/0.0231 **0.220**	0.0062/0.0223 **0.279**	0.0044/0.0255 **0.173**
αCENH3 *S*.*strictum*			0.0072/0.0221 **0.325**	0.0052/0.0195 **0.267**
**(B)**				
	**βCENH3** ***S***. ***strictum***		**βCENH3** ***S***. ***sylvestre***	
	**NTT**	**HFD**	**NTT**	**HFD**
βCENH3 *S*. *cereale*	0.0144/0.0430 **0.335**	0.0077/0.0371 **0.208**	0.0198/0.0411 **0.482**	0.0068/0.0339 **0.201**
βCENH3 *S*. *strictum*			0.0179/0.0335 **0.534**	0.0056/0.0348 **0.162**

**A** - αCENH3; **B** – βCENH3. Bold numbers indicate the ratio ω = K_a_:K_s_.

Even though the full-length NTT and HFD sequences reveal signatures of stabilizing selection, it is still possible that some of the sites within these domains have been under other modes of selection. Supplementary Table S2 summarizes the characteristics of these variable codons, which occur in at least several accessions, that is, their variability is not accounted for by random effects or by sequencing errors. Codon 34 containing only nonsynonymous substitutions in the αNTT of the annual subspecies *S*. *dighoricum*, *S*. *cereale ssp*. *ancestrale* (*S*. *ancestrale* throughout) and the perennial subspecies *S*. *anatolicum* is under diversifying selection. Codons 22 and 48 in the αNTT and codons 92 and 136 in the αHFD, as well as just one substitution at codon 51 in the βNTT as being under negative selection. Synonymous substitutions occur at these codons in all the *S*. *cereale* and *S*. *strictum* subspecies. Noteworthy, all the accessions with codons under diversifying or negative selection are cross-pollinated. Variable codons that are not undergoing selection occur most frequently in the most ancient annual self-pollinated *S*. *sylvestre*. Thus, diversifying selection operates at a very few sites of the N-terminal tail of αCENH3 and the HFD of βCENH3 of cross-pollinated species and adds little to the structural diversity of the existing forms of CENH3 proteins.

### Divergence of CENH3 histone fold domains in Triticeae

Considering an important functional role of the HFD, which is crucial for nucleosome assembly and targeting of CENH3 to centromeres^23^, we extended the analysis of its structure to *Triticum*, the genus most closely related to *Secale*, and species progenitors to polyploid wheat species (Table 1).

Two types of CENH3s had previously been identified in diploid and tetraploid wheat species^13^. The HFD sequences of *Triticum*, *Aegilops* and *Secale* accessions are shown on Fig. 3. In *Triticum* and *Aegilops*, the C-terminal part of *ßCENH3* is distinct from that of *αCENH3* in that (a) some of its positions are polymorphic, which leads to synonymous and nonsynonymous amino acid substitutions, and (b) it has the asparagine-encoding trinucleotide АAС inserted near the top of loop 1.

In diploid ancestors, the main HFD forms appear in two variants, having specific amino acids at particular positions, one in the αHFD (not shown) and six in the βHFD (asterisked in Fig. 3). The amino acid sequences of the HFDs of allopolyploid wheats display no synonymous substitutions. In rye αHFD sequences have the highest level of similarity in both between v-1, v-2 variants and between species. The specific amino acids that make the rye species distinct from all *Triticum* accessions occupy only six positions: one in the αHFD (in loop N) and five in the βHFD, four of which are in the САТD (Fig. 3). Importantly, four out of five amino acids occur in βHFD-v1, suggesting that was the preferred beta form variant during *Secale* evolution. Comparisons for βHFD-v2 revealed signatures of positive selection in the genomes of allopolyploid wheat species (Table 3). However, according to Fisher’s exact test, ω values >1 in these cases failed to reach significance because the K_a_/K_s_ ratio was just slightly higher between than within species. All the ω values for *ßCENH3* sequences in the *H*. *vulgare* and *H*. *bulbosum* genomes are similar to their counterparts in wheat and rye species; however, the absolute values of K_a_ and K_s_ are several times higher. Noteworthy, the differences are more salient for the cultivated barley *H*. *vulgare* (Table 3).

**Тable 3:** Nonsynonymous (K_a_) to synonymous (K_s_) nucleotide substitutions in the βHFD of CENH3 of various Triticeae species.

	CENH3 *T. durum*	CENH3 *T. aethiopicum*	CENH3 *T. aestivum*	CENH3 *T. compactum*	CENH3 *S. sylvestre*	CENH3 *S. cereale* (cv. Otello)	CENH3 *S. strictum*	CENH3 *S. kuprijanovii*
βCENH3-v1*	0.0334/0.0700.477	0.0313/0.07390.423	0.0249/0.06310.395	0.0249/0.08560.290	0.0333/0.04510.737	0.0352/0.04100.859	0.0294/0.09640.305	0.0339/0.05150.658
βCENH3-v2*	0.0272/0.0066**4.12**	0.0251/0.0099**2.53**	0.0187/0.000—	0.0187/0.02010.930	0.0585/0.06970.839	0.0598/0.06550.913	0.0544/0.12190.446	0.0594/0.07520.790
βCENH3 of H. bulbosum**	0.1727/0.31130.555	0.1659/0.31730.523	0.1621/0.30210.537	0.1625/0.29990.542	0.1467/0.28070.523	0.1497/0.28630.523	0.1503/0.32690.460	0.1491/0.29160.511
βCENH3 of H.vulgare**	0.2396/0.24200.701	0.2339/0.34290.682	0.2276/0.33430.681	0.2273/0.33590.677	0.2178/0.30500.714	0.2215/0.30630.723	0.2137/0.36230.590	0.2165/0.31840.680

*These sequences were taken from *T*. *urartu, A*. *tauschi*, and *A*. *speltoides*. **JF419330.1 and JF419329.1. Figures in bold are K_a_/K_s_ values higher than 1.

### Alternative splicing of ScCENH3s

**Figure 4:**
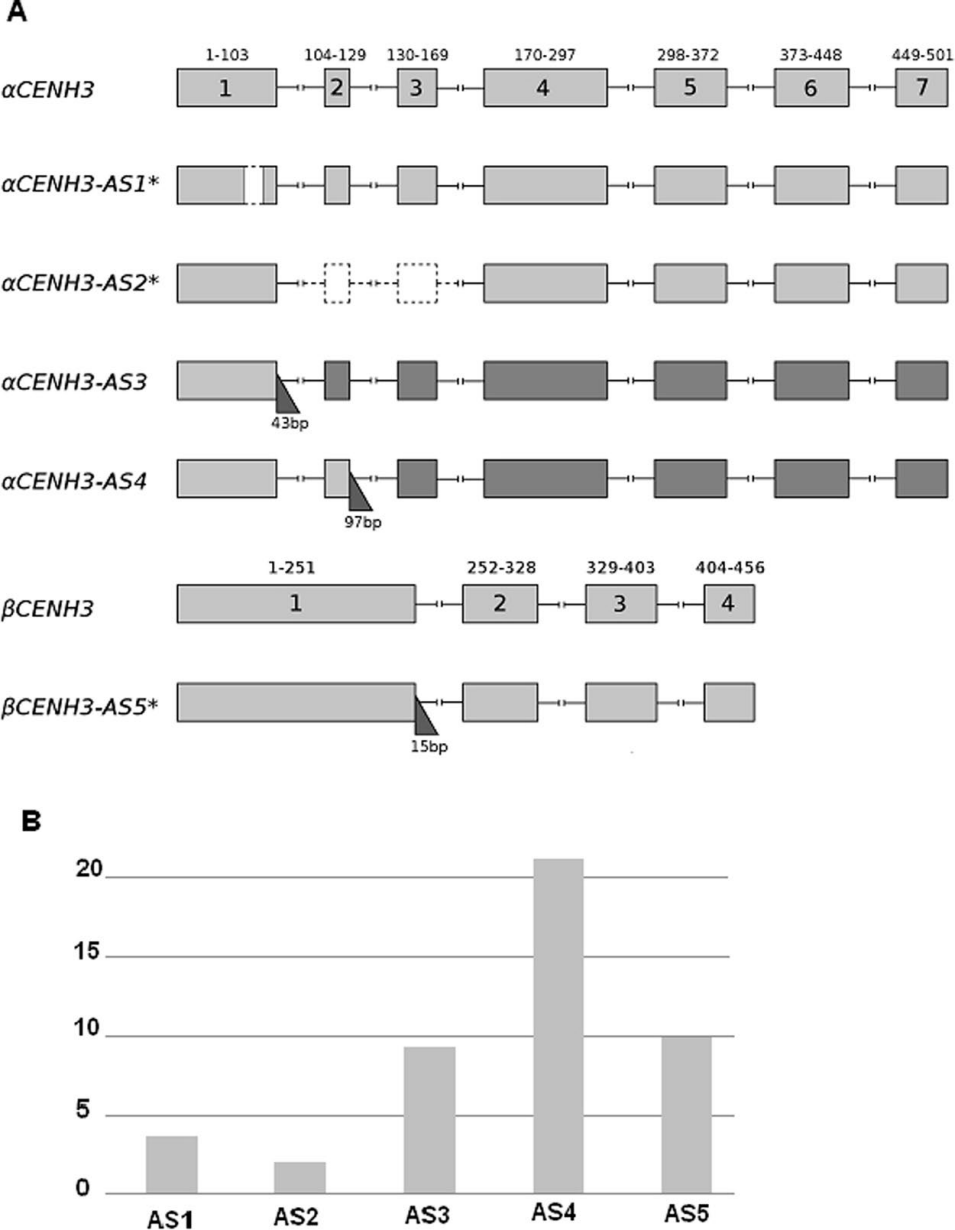
Intron-exon structure of CENH3 genes in *S*.*cereale*. (**A**) Schematic of splicing isoforms. Exons are enumerated and are depicted as light gray rectangles; introns are depicted as black lines connecting exons; the ranges on top of exons indicate exon boundaries. Introns are not to scale. Deletions are depicted as dashed rectangles. Right angled triangles point to retained intron fragments, with their sizes as indicated below. Putatively functional forms with conserved HFD structure are asterisked. Exons with reading frame shifts due to intron retention events are depicted as dark gray rectangles. (**B**) Percentage of frequency of occurrence of splicing isoforms in rye accessions. AS1-AS4 isoforms expressed as a percentage of the total αCENH3 NTT clones. AS5 isoform expressed as a percentage of the total βCENH3 HFD clones.

Alignment of the cDNA sequences of α*ScCENH3* and β*ScCENH3* with publically available genomic rye sequences^24^ confirmed that the open reading frame of α*ScCENH3* comprises 7 exons and 6 introns, while that of β*ScCENH3*, 4 exons and 3 introns (Fig. 4А). Exons 2 and 3 of *αScCENH3* are very short, 25 bp and 39 bp, respectively; exon 4 of β*ScCENH3* is 52 bp in length. In addition to the main *ScCENH3* forms, we obtained several variants of transcripts which will be referred to as alternative splicing (AS) isoforms throughout. The N-terminal domain of the α*ScCENH3* gene is the source of most AS products. The following isoforms were identified: (1) *ScCENH3*-AS1 flanked by splice sites and containing a 21-bp deletion in exon 1 from position 67 to position 87; (2) *ScCENH3*-AS2 flanked by splice sites and containing a 66-bp deletion, which removes exon 2 (104–129 nt) and exon 3 (130–169 nt) entirely; (3) *ScCENH3*-AS3, with retention of a 43-bp fragment of intron 1 after position 103; and 4) *ScCENH3*-AS4, with retention of a 97-bp fragment of intron 2 after position 129. These isoforms are consistent with the main types of alternative splicing^25^: *ScCENH3*-AS1 appears due to the proximity of two splice sites; *ScCENH3*-AS2, due to mutually exclusive exons; *ScCENH3*-AS3 and *ScCENH3*-AS4, due to intron retention.

Only the most frequent isoform AS4 occurs in all *Secale* accessions. For example, isoform AS2 was found in annual *S*. *segetale* and rye cultivars Otello and Imperial (2.8% of all α*ScCENH3* transcripts, Fig. 4B), but not in perennial species. Noteworthy, although the 21-bp and 66-bp deletions shorten the N-terminal domain, they do not cause a shift in the open reading frame – nor do they modify the amino acid sequence of the HFD of αCENH3. The 21-bp deletion contains CAG, a false acceptor site.

β*ScCENH3* was found to contain only one AS isoform (retention of intron 1). It has an unusual localization in the HFD (Fig. 4A). The 15-bp insertion is before loop 1 and has therefore no effect on the βHFD CATD structure. Thus, the protein sequences of AS isoforms have the same CATD structure as the main ScCENH3 forms and can potentially participate in the formation of centromeric nucleosomes, thus increasing the diversity of rye CENH3 proteins.

## Discussion

We have identified two distinct forms of *CENH3* transcripts, α*CENH3* and β*CENH3*, in 11 rye species and subspecies. This finding indicates that the rye genome contains at least two copies of the *CENH3* gene. The closest relatives of rye–wheat, *Aegilops* and barley–had previously also been found to have two main forms of CENH3^13,14^. In barley, these copies are encoded by two different chromosomes^14^. Thus, the presence of these two forms in *Triticeae* appears to be one of the features of this tribe. Both copies avoided elimination from the rye genome, even though elimination by massive local deletions is often imminent after duplications^26^. This strongly suggests that both CENH3 paralogs are required for proper centromere function in Triticeae. On the other hand, functional inactivation of β*CENH3* did not result in an obvious somatic phenotype in barley^27^.

Alpha CENH3s cluster with CENH3 of *О*. *sativa*, a precursor of the Triticeae species, encoding only one variant of CENH3, therefore it is likely that the alpha forms of Triticeae species appeared earlier than the beta forms. The K_a_ and K_s_ values as well as their ratios between the beta forms of CENH3 are higher than the corresponding values for the alpha forms (Table 2, Supplementary Table S1), as if the beta forms were under relaxed selective constraints, which result in elevated rates of evolution. A similar tendency is observed in the genus *Triticum*, in which the αCENH3 HFDs are under negative (stabilizing) selection and the βCENH3 HFDs are undergoing positive (adaptive) evolution^13^.

The amino acid sequences of CENH3 in the rye species and subspecies display a surprisingly high level of similarity, despite the differences that they have in morphology, life-cycle duration and pollination systems as well as environmental and growing conditions. The highest number of amino acid differences was observed in the βCENH3 of the most ancient species, *S*. *silvestre*, but only two of them were found to be nonsynonymous substitutions (Supplementary Table S2). Low ω values obtained from most of the pair-wise comparisons (Table 2, Supplementary Table S1) suggest that *Secale CENH3* appears to have evolved under strong purifying selection. *Triticum* also exhibits the evolutionary conservatism of the CENH3 structure (Table 3), which is even more pronounced in polyploid wheat species. The complete structure of the alpha and beta forms of CENH3 was determined in the cultivated *H*. *vulgare* and its close wild relative *H*. *bulbosum* ^14^. A comparison of these two species reveals the heterogeneity of CENH3 structure, which contrasts with the homogeneity of this protein in *Secale* and is even higher than the genus-specific differences between *S*. *cereale* and *T*. *aestivum*.

Comparison of the dynamics of evolutionary changes occurring in the CENH3s of *Hordeum*, *Secale* and *Triticum* leads to the assumption that the structure of this protein evolved at different rates in these genera. Considerable differences between the *Hordeum* species suggest that this genus is the only one among the three that have rapidly evolving CENH3 genes. What factors could possibly account for different rates of structural changes in CENH3 within closely related genera of a tribe? According to by far the most extensive molecular marker-based study, most *Hordeum* species are not older than 2 MY^28^. The rye/wheat split time is estimated at approximately 3.5–4 MYA^29^ and 3–9 MYA^30^. Later on, the genome donors of hexaploid wheat split off between 2.1–2.9 MYA^29^ and the age of the *Secale* crown group is estimated at 1.7 MY^31^. Thus, the existing estimates of split times for *Hordeum*, *Secale* and *Triticum* species suggest that age alone is unlikely to be a factor that accounts for such strong differences in the rates of evolutionary changes in CENH3 structure between the barley species on the one hand and the *Secale/Triticum* complex on the other.

Hybridization and associated introgression of genetic material are powerful evolutionary factors, and remote hybridization played an important role in plant speciation. Compared to *Hordeum*, the genus *Secale* consists of much fewer taxa, most of which are cross-pollinated. Most rye species and subspecies cross readily with each other and with cultivated rye and produce vigorous hybrids with completely normal meiosis and high pollen fertility^32^, suggesting the absence of reproductive barriers^33^. Indeed, a genome-wide comparative analysis showed that the rye genome represents a concatenation of genomic segments of different evolutionary origin and is likely to have been shaped by introgressive hybridization or reticulate evolution^23^. In support based on the work by Escobar and the co-workers^34^ *Hordeum* species follow a tree-like pattern of evolution, while *Secale*, *Triticum*, *Aegilops* are more reticulated than any other clade. Thus, our data favor the assumption that the process of genome formation for *Secale* was accompanied by ancestral hybridization events. It appears that such reticulate evolution served as a factor stabilizing the structure of the CENH3 genes and proteins, and this factor was more powerful within *Secale* and *Triticum* than it was in the other taxa, including *Hordeum*.

Three splicing isoforms were found among the rye subspecies that do not disrupt the CATD structure: the 21- and 66-bp deletions in the αNTT were largely found in the annual species, and the 15-bp insertion, in the βHFD (Fig. 4). Alternative splicing at the C-terminus in rye does not affect the structure of the DNA-binding domain, but can influence CENH3 binding to other kinetochore proteins, for example, CENP-C, which, in addition to being a DNA-binding protein, can bind to the C-terminal tail of CENP-A^35^.

Thus, the factors responsible for CENH3 diversity in the rye species are (1) the occurrence of *CENH3* in two forms, α*CENH3* and β*CENH3*, with two variants of each of these forms, and (2) the products of alternative splicing, which are presumably driven by positive selection^36^. In wheat, 95% of alternative transcripts from a particular gene exhibited different expression profiles, as was revealed by a hierarchical clustering of 30,232 transcripts^37^. It appears that AS isoforms have complementary functions, thereby enhancing the adaptation-related potential of proteins. This finding is indicative of an evolutionary stability and conservation of the genetic factors that control the CENH3 structure in the genus *Secale*.

## Methods

### Plant material and plant growth

We selected 13 accessions of weedy/wild rye and cultivated rye representing the most commonly recognized taxa ranked as species or subspecies in the genus *Secale* according to Sencer and Hawkes^17^ (Table 1). Seeds were kindly provided by the Leibniz Institute of Plant Genetics and Crop Plant Research (Germany), the United States Department of Agriculture (USA) and N.I.Vavilov Research Institute of Plant Industry (Russia) from their respective germplasm collections. *Triticum* and *Aegilops* seeds were kindly provided by Dr. B. Kilian. Accessions are listed and characterized in Table 1. All plants were grown in a greenhouse at 22 °C: 18 °C, day: night with a 16-h day length.

### Screening of databases

Pyrosequenced rye cDNAs (GABI-RYE EXPRESS project, accession: PRJEB2219 ID:203975) from the NCBI SRA database (http://www.ncbi.nlm.nih.gov/sra/) were analyzed using the WUBLAST software (http://blast.wustl.edu) and reads with high similarity to αCENH3 of *H*. *vulgare* (JF419328.1) and βCENH3 of *T*. *urartu* (KM507184) were revealed. These reads were used for generation of rye αCENH3 and βCENH3 contigs by the CodonCodeAligner software program (http://www.codoncode.com/aligner). The search for rye genomic CENH3 sequences was performed in the DNA database (European Bioinformatics Institute sequence read archive, accession ID ERP001745) obtained from sorted rye chromosomes 1R-7R^24^.

### RNA isolation and PCR amplification

Total RNA was isolated from leaves of 12-day-old seedlings using the TRI Reagent (MRC, Inc., USA) and treated by RQ-RNase-Free DNase (Promega, Madison, WI) according to the manufacturer’s instructions. RNA was reverse-transcribed to cDNA using a RevertAid H Minus First Strand cDNA Synthesis Kit (Thermo Fisher Scientific). Specific primers used to amplify *CENH3* and its domains, NTT and HFD, from rye cDNA are presented in Supplementary Table S3. For amplification of HFD *CENH3* from *Triticum* and *Aegilops* species, we used a set of degenerated primers designed for monocotyledons^38^.

### Sequencing and sequence alignment

RT-PCR products were purified using a Qiagen Purification Kit (Qiagen) and cloned using an InsTAclone PCR Cloning Kit (Thermo Fisher Scientific). Both strands of 12–20 clones of each accession were sequenced using an ABI 3130 × 1 Genetic Analyzer (Applied Biosystems Inc., CA) and an ABI BigDye Kit according to a standard protocol. Similarity searches between the obtained rye *CENH3* sequences and their orthologous from other species were carried out using the TBLASTN software^39^ in the NCBI database (http://blast.ncbi.nlm.nih.gov/Database/). Multiple alignments of amino acids and coding sequences were performed online using Clustal Omega^40^ (http://www.ebi.ac.uk/Tools/msa/clustalo). Alignments were further refined manually and used for downstream analysis with the aid of statistical, phylogenetic programs and for visualization^41^ (http://www.jalview.org). The deduced protein sequences were examined for potential posttranslational modifications using NetPhos 2.0 (www.cbs.dtu.dk/services/NetPhos).

### Phylogenetic analysis, tests for positive selection

Phylogenetic trees were drawn using MEGA6^42^. Mean pairwise amino acid and nucleotide distances were also calculated using MEGA6 according to the Poisson and T92 + G models. Bootstrap values were calculated from at least 1,000 replications.

Sequences were analyzed for deviations from neutrality with the McDonald–Kreitman^22^ test using DnaSP^43^. Analysis of the ratios of nonsynonymous (K_a_) to synonymous (K_s_) substitutions (ω) was performed using DnaSP. The statistical significance of positive selection was calculated by Fisher’s exact test as implemented in MEGA6. MEME and SLAC (with a significance level cutoff of 0.05 and 0.1, correspondingly) analyses were performed through the Datamonkey server (http://datamonkey.org/).

### Data availability

The sequence data described are available in GenBank under accession numbers MG384763–MG384788.

## Electronic supplementary material

Supplementary information
