# Supplementary material for: Conserved molecular structure of the centromeric histone CENH3 in Secale and its phylogenetic relationships
Source: Sci Rep. 2017 Dec 15;7:17628. doi: 10.1038/s41598-017-17932-8 (PMC5732303; doi:10.1038/s41598-017-17932-8)
Supplement: Supplementary file 1 — Supplementary information [file 41598_2017_17932_MOESM1_ESM.pdf]

Supplementary Information

# Conserved molecular structure of the centromeric histone CENH3 in *Secale* and its phylogenetic relationships

E.V.Evtushenko[1], E.A.Elisafenko[2], S.S.Gatzkaya[1], Y.A.Lipikhina[1], A.Houben[3], A.V.Vershinin[1*]

[1] Institute of Molecular and Cellular Biology SB RAS, Novosibirsk, 630090, Russia

[2] Institute of Cytology and Genetics SB RAS, Novosibirsk, 630090, Russia

[3] Leibniz Institute of Plant Genetics and Crop Plant Research (IPK), Gatersleben, 06466 Stadt Seeland, Germany

* corresponding author: avershin@mcb.nsc.ru

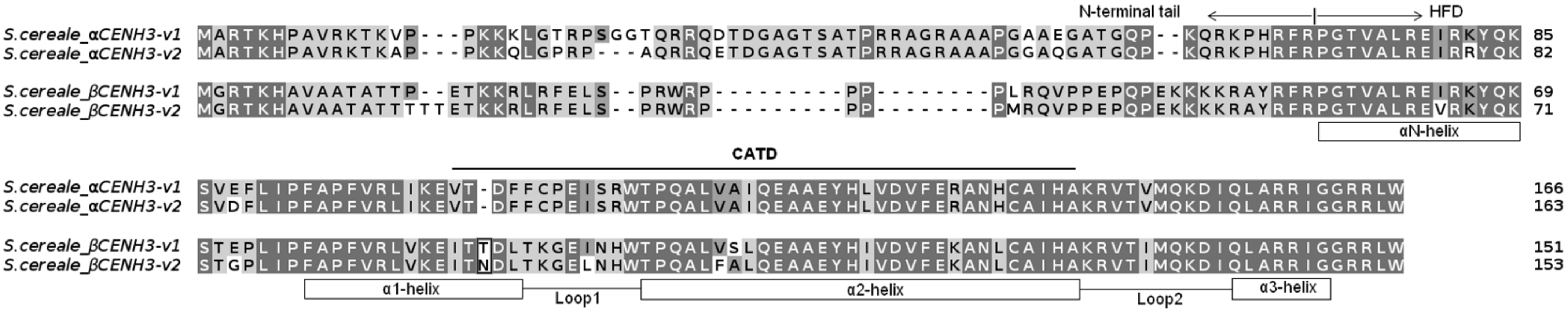


**Supplementary Fig. S1. Multiple alignment of the amino acid sequences of CENH3 variants in *S. cereale* (variety "Imperial")**
The amino acids that occur in the βCENH3 HFD, but not in the αCENH3, are framed.

**Supplementary Table S1. Nonsynonymous ($K_a$) to synonymous ($K_s$) nucleotide substitutions in the NTT domain of βCENH3s of *Secale* subspecies**

| | CENH3 *S. cereale* | CENH3 *S.afghanicu* | CENH3 *S.vavilovii* | CENH3 *S. strictum* | CENH3 *S.kuprijanov* | CENH3 *S.africanum* |
|---|---|---|---|---|---|---|
| CENH3 *S.sylvestre* | 0.022/0.048<br>0.457 | 0.018/0.027<br>0.668 | 0.020/0.048<br>0.421 | 0.017/0.029<br>0.579 | 0/020/0.048<br>0.420 | 0.017/0.026<br>0.660 |
| CENH3 *S.cereale* | | 0.020/0.062<br>0.322 | 0.0009/0.0064<br>**1.48** | 0.019/0.066<br>0.291 | 0.0092/0.009<br>0.984 | 0.019/0.064<br>0.309 |
| CENH3 *S.afghanicu* | | | 0.012/0.063<br>0.296 | 0.014/0.021<br>0.537 | 0.019/0.063<br>0.296 | 0.010/0.017<br>0.581 |
| CENH3 *S.vavilovii* | | | | 0.017/0.067<br>0.266 | 0.007/0.003<br>**2.07** | 0.018/0.066<br>0.279 |
| CENH3 *S.strictum* | | | | | 0.018/0.067<br>0.265 | 0.008/0.017<br>0.515 |
| CENH3 *S.kuprijanovii* | | | | | | 0.018/0.066<br>0.278 |

Bold numbers indicate the ratio $\omega = K_a : K_s > 1$

**Supplementary Table S2. Description of amino acid substitutions in CENH3 sequences**

| # | CENH3 domain | Codon | Species, subspecies | Codon substitution | Amino acid substitution | Substitution Character | P-value | Significance level | Method |
|---|---|---|---|---|---|---|---|---|---|
| 1* | αCENH3 *v-1*, NTT | 34* | *S. ancesrale* (AO)<br>*S. dighoricum* (AO)<br>*S. anatolicum* (PO) | ACA→ GCA<br>ACA→ CCA<br>ACA→ CCA | T→A<br>T→P<br>T→P | non-synonymous | 0.0433 | 0.05 | MEME |
| 2* | αCENH3 *v-1*, NTT | 22 | *S. cereale* (AO, AS) | ACG→ACC | no change (T) | synonymous | 0.0123 | 0.1 | SLAC |
| 3* | αCENH3 *v-1*, NTT | 48 | *S. strictum* (PO, PS) | CGT→CGG | no change (R) | synonymous | 0.0557 | 0.1 | SLAC |
| 4* | αCENH3 *v-1*, HFD | 92 | *S. cereale* (AO, AS) | CCT→CCC(G) | no change (P) | synonymous | 0.0370 | 0.1 | SLAC |
| 5* | αCENH3 *v-1*, HFD | 136 | *S. strictum* (PO, PS) | AGG→CGG | no change (R) | synonymous | 0.0017 | 0.1 | SLAC |
| 6* | βCENH3 *v-1*, NTT | 51 | *S. strictum* (PO)<br>*S. afghanicum* (AO) | GCG→GCT<br>GCG→GCT | no change (A) no change (A) | synonymous | 0.0370 | 0.1 | SLAC |
| 7 | αCENH3 *v-1*, NTT | 10 | *S. cereale* (AO) (10)**<br>*S. strictum* (PO) (33)<br>*S. anatolicum* (PO) (40)<br>*S. sylvestre* (AS) (14) | AGG→GGG | R→G | non-synonymous | _ | _ | _ |
| 8 | αCENH3 *v-1*, NTT | 31 | *S. strictum* (PO) (11)<br>*S. anatolicum* (PO) (5)<br>*S. sylvestre* (AS) (7) | CGG→GGG | R→G | non-synonymous | _ | _ | _ |
| 9 | αCENH3 *v-1*, NTT | 33 | *S. cereale* (AO) (22)<br>*S. strictum* (14)<br>*S. sylvestre* (AS) (7) | GAC→GAG | D→E | non-synonymous | _ | _ | _ |
| 10 | αCENH3 *v-1*, HFD | 104 | *S.strictum* (PO) (20)<br>*S.anatolicum* (PO) (10)<br>*S.africanum* (PS) (10) | ACC→GCC | T→A | non-synonymous | _ | _ | _ |
| 11 | αCENH3 *v-1*, HFD | 110 | *S.strictum* (PO) (18)<br>*S.anatolicum* (PO) (11)<br>*S.africanum* (PS) (5) | GAA→AAA | E→K | non-synonymous | _ | _ | _ |
| 12 | αCENH3 *v-1*, HFD | 130 | *S.strictum* (PO) (12)<br>*S.anatolicum* (PO) (11)<br>*S.africanum* (PO) (14) | CTC→CAC | L→H | non-synonymous | _ | _ | _ |
| 13 | βCENH3 *v-1*, NTT | 17 | *S.sylvestre* (AS) (80) | AAG→ACG | K→T | non-synonymous | _ | _ | _ |
| 14 | βCENH3 *v-1*, NTT | 41 | *S.sylvestre* (AS) (67) | GAG→CAG | E→Q | non-synonymous | _ | _ | _ |

* CENH3 sequences are under selective pressure

** relative number of substitution (%)

**Supplementary Table S3. List of primers used for amplification of full-length sequences and separate domains of CENH3**

| Primer number | Primer Sequence, 5´ – 3´ | Amplified sequence of *CENH3* |
|---|---|---|
| 1<br>2 | ATGGCCCGCACCAAGCAC<br>GCATCACCAAAGCCTCC | Coding part of the rye *αCENH3* gene |
| 3<br>4 | TGGGTCGCACGAAGCAC<br>TCACCAAAGCCTTCTCCCC | Coding part of the rye *βCENH3* gene |
| 5 | GAAACTCGACCGACTTCTG | NTT of the rye *αCENH3* gene (with primer #1) |
| 6<br>7 | ATGGGTCGCACGAAGCAC<br>CGTGAGGTCGGTGGTGATTT | NTT of the rye *βCENH3* gene |
| 8 | GTGGCACTGCGGGAGATCAGGA | HFD of the rye *αCENH3* gene (with primer #2) |
| 9 | GCGCTGCGGGAGATCAG | HFD of the rye *βCENH3* gene (with primer #7) |
| 10<br>11 | GTRGCRCTGCGGGAGATCAGGA<br>CTBGCRAGYTGYATGTCCTTTT | HFD of the *CENH3* gene from *Triticum* and *Aegilops* species |
